# Supplementary material for: Does culture moderate the relationships between rumination and symptoms of posttraumatic stress disorder and depression?
Source: PLoS One. 2022 Nov 29;17(11):e0278328. doi: 10.1371/journal.pone.0278328 (PMC9707787; doi:10.1371/journal.pone.0278328)
Supplement: Attachment — Submitted filename: PONE-D-22-19386 research article commented.docx [file pone.0278328.s001.docx]

**Comments**

Clarifying the lack in scientific community regarding cultural influence in the associations between brooding rumination and symptoms of depression and PTSD, is a very important research to fill the existing gap in the research world. The author has raised a good topic but the manuscript does not follow the necessary findings. The introduction, methodology, result, discussion and conclusion section need to be scientifically written. Some major revisions are required before it could be considered for publication as follows:

1. I have doubt regarding the generalizability of your study to other world communities from different cultural background. The study may be failed when we think of other communities and how do you manage this? I mean do you think that cultural group did not moderate the relationships between brooding rumination and symptoms of depression and PTSD for different communities from different cultural background? It should be limitation for this study because you wrote that accumulating research suggests that the relationship between brooding rumination and psychopathology may vary between different cultural groups.
2. Authors are required to re-write the abstract. Authors must show how study participants were selected, how data were analyzed and the study good results in the abstract section to enhance the readers.
3. Please concrete the keywords and make them formally and academically. You have repeated “PTSD; post-traumatic stress disorder;”, so try to write/use only one of them.
4. Authors must check the manuscript carefully before submitting it to the journal. Because still manuscript having grammar mistake and spellings mistake. English language needs polishing throughout the manuscript. Have it edited by a professional.
5. To avoid any plagiarism and integrate the concepts from different researches of your interest, please give references at the end of each sentence for the paragraph “However, this evidence base is almost entirely derived from Western samples. Consequently, the dominant paradigm that suggests rumination is universally associated with increased psychopathology, is a notion based largely on Western research and cultural values. This is a key limitation given that (1) globally, many individuals with depression and PTSD do not come from Western cultural backgrounds, and (2) accumulating research suggests that the relationship between brooding rumination and psychopathology may vary between different cultural groups.”
6. You have described that brooding rumination has been found to be less associated with poor psychological outcomes, including anxiety, depression and life dissatisfaction, in Asian individuals compared to Western individuals. So, what you would do if your study might come with no association?
7. You have tried to address three hypotheses, but your title only focuses on the cultural influence. What about the other two hypotheses “brooding rumination would be positively associated with both depression and PTSD symptom severity” and “there would be greater brooding rumination reported by the Malaysian group than the Australian group?
8. I think it is better to say something in the method section as a “study area” about Australian and Malaysian, their culture, prevalence of brooding rumination, depression and PTSD symptom as characteristics of the community may be from other studies.
9. You have used convenience sampling method, and do you think your study is representative to the general population of Australian and Malaysian who have brooding rumination? If not, what is the significance of the study?
10. Do you think 253 study participant is enough to say a research for such large populated countries? It is better to increase your sample size which may affect your current result.
11. Try to clearly explain the following criteria in your document, why age between 18 and 65?
12. Please provide any reference for this “PTSD Checklist for DSM-5 (PCL-5) with Life Events Checklist for DSM-5 (LEC-5)” from previously conducted studies on this area.
13. If you have ethical approval obtained from BLINDED, please try to attach.
14. How did you assured that the data you collected have good quality? Are you confident for saying I had good research tool because of its co-design by Australian and Malaysian researchers?
15. It there any other additional information you collected from participants before the study begins to check whether they are real participants who fulfilled the criteria or not? helpful to control biases.
16. You have not described the result of the study in detail. First, prepare one table independently for socio-demographic characteristics. Try to describe the socio-demographic characteristics of the participant’s independently in one table and discuss within one or two paragraph about the result in the table. You have to describe the result of your current tables’ one and two in detail within at least each two paragraphs above the tables.
17. If you did not find a significant moderating effect of cultural group on the relationship between brooding rumination and either depression or PTSD symptom severity, what you would recommend in relation to other researches that reported culture influence in the association?
18. **Our finding that** Malaysians reported higher levels of brooding rumination compared to Australians aligns with previous cross-cultural non-clinical research; members of collectivistic cultures (e.g., Malaysia) tend to brood more than members of individualistic cultures (e.g., Australia). What is your justification for this finding or reason?
19. It is better to separate your discussion section into two separate sections “Discussion, and conclusion”. Discuss you conclusion with recommendations?
20. The discussion is too short, so it needs both detail internal and external discussions with clear justifications.
21. You have a lot of limitation, and I think it will have effect on the acceptability of you result for publication. So, try to reduce limitations and display only core limitations in your discussion section.
22. Please try to include abbreviation section and authors’ contribution section at the end.
23. Try to number and heading the titles and subtitles of your document starting from introduction.
