# Supplementary material for: Does culture moderate the relationships between rumination and symptoms of posttraumatic stress disorder and depression?
Source: PLoS One. 2022 Nov 29;17(11):e0278328. doi: 10.1371/journal.pone.0278328 (PMC9707787; doi:10.1371/journal.pone.0278328)
Supplement: Attachment — Submitted filename: Paper-reviewed.docx [file pone.0278328.s002.docx]

**PLOSONE**

**DoesCultureModeratetheRelationshipsbetweenRuminationandSymptomsofPosttraumaticStressDisorderandDepression?**

--ManuscriptDraft--

| **ManuscriptNumber:** | PONE-D-22-19386 |
| --- | --- |
| **ArticleType:** | ResearchArticle |
| **FullTitle:** | DoesCultureModeratetheRelationshipsbetweenRuminationandSymptomsofPosttraumaticStressDisorderandDepression? |
| **ShortTitle:** | ExaminingculturaldifferencesinruminationindepressionandPTSD |
| **CorrespondingAuthor:** | BryanLee,BPsych(Hons)  UniversityofMelbourne-ParkvilleCampus:UniversityofMelbourneMelbourne,VictoriaAUSTRALIA |
| **Keywords:** | Rumination;depression;PTSD;post-traumaticstressdisorder;brooding;emotionregulation;cross-culturaldifferences |
| **Abstract:** | Brooding rumination is positively associated with symptoms of both depression andposttraumatic stress disorder (PTSD). However, non-clinical cross-cultural researchindicatesthatculturemayinfluencetheseassociations.Thisstudyaimedtoexaminethe moderating effect of cultural group (Australian versus Malaysian) on theassociations between brooding rumination and symptoms of depression and PTSD.European Australians (n= 109) and Malaysians of varying Asian heritages (n= 144)completed an online questionnaire containing the Hospital Anxiety and DepressionScale, PTSD checklist for DSM-5 and the Ruminative Response Scale-Short Form.First, Malaysian participants had higher brooding rumination than Australianparticipants. Second, higher levels of brooding rumination were positively associatedwith depression and PTSD symptom severity. Third, contrary to our expectations,cultural group did not moderate the relationships between brooding rumination andsymptoms of depression and PTSD. If replicable, these results suggest that existingassessment and treatment approaches that target brooding rumination may apply toMalaysianindividualswithdepressionandPTSD. |
| **OrderofAuthors:** | BryanLee,BPsych(Hons) |
|  | HaoxiangLi |
|  | TamsynReyneke |
|  | ShamsulHaque |
|  | SitiZainabAbdullah |
|  | BritneyKerrWenTan |
|  | BelindaLiddell |
|  | LauraJobson |
| **AdditionalInformation:** |  |
| **Question** | **Response** |
| **FinancialDisclosure**  Enterafinancialdisclosurestatementthatdescribes the sources of funding for thework included in this submission. Reviewthe [submission guidelines](http://journals.plos.org/plosone/s/submission-guidelines#loc-financial-disclosure-statement)for detailedrequirements. View published researcharticles from [*PLOS ONE*](http://journals.plos.org/plosone/)for specificexamples. | Theauthorsreceivednospecificfundingforthiswork. |

| This statement is required for submissionand **will appear in the published article** ifthesubmissionisaccepted.Pleasemakesureitisaccurate. |  |
| --- | --- |
| **Unfundedstudies**  Enter:*Theauthor(s)receivednospecificfundingforthiswork.*  **Fundedstudies**  Enterastatementwiththefollowingdetails:   - Initialsoftheauthorswhoreceivedeachaward - Grantnumbersawardedtoeachauthor - Thefullnameofeachfunder - URLofeachfunderwebsite - Didthesponsorsorfundersplayanyroleinthe study design, data collection andanalysis, decision to publish, or preparationofthemanuscript? - **NO** - Include this sentence at the end ofyourstatement:*Thefundershadnoroleinstudy design, data collection and analysis,decision to publish, or preparation of themanuscript.* - **YES**-Specifytherole(s)played. |  |
| *typeset |  |
| **CompetingInterests**  Use the instructions below to enter acompeting interest statement for thissubmission. On behalf of all authors,disclose any [competing interests](http://journals.plos.org/plosone/s/competing-interests)thatcould be perceived to bias thiswork—acknowledgingallfinancialsupportand any other relevant financial or non-financialcompetinginterests.  This statement is required for submissionand **will appear in the published article** ifthe submission is accepted. Please makesure it is accurate and that any fundingsourceslistedinyourFundingInformationlater in the submission form are alsodeclared in your Financial Disclosurestatement.  Viewpublishedresearcharticlesfrom  [*PLOSONE*](http://journals.plos.org/plosone/)forspecificexamples. | Theauthorshavedeclaredthatnocompetinginterestsexist. |

| **NOauthorshavecompetinginterests**  Enter:*Theauthorshavedeclaredthatnocompetinginterestsexist.*  **Authorswithcompetinginterests**  Entercompetinginterestdetailsbeginningwiththisstatement:  *I have read the journal's policy and theauthorsofthismanuscripthavethefollowingcompeting interests: [insert competinginterestshere]* |  |
| --- | --- |
| *typeset |  |
| **EthicsStatement**  Enter an ethics statement for thissubmission.Thisstatementisrequiredifthestudyinvolved:   - Humanparticipants - Humanspecimensortissue - Vertebrateanimalsorcephalopods - Vertebrateembryosortissues - Fieldresearch   Write"N/A"ifthesubmissiondoesnotrequireanethicsstatement.  General guidance is provided below.Consult the [submission guidelines](http://journals.plos.org/plosone/s/submission-guidelines#loc-guidelines-for-specific-study-types)fordetailed instructions. **Make sure that allinformationenteredhereisincludedintheMethodssectionofthemanuscript.** | ThestudyobtainedethicalapprovalfromtheMonashUniversityHumanResearchEthics Committee (2021-27577-58747). Written consent was obtained from allparticipantsinvolvedinthestudy. |

| **Formatforspecificstudytypes**  **HumanSubjectResearch(involvinghumanparticipantsand/ortissue)**   - Give the name of the institutional reviewboardorethicscommitteethatapprovedthestudy - Includetheapprovalnumberand/orastatement indicating approval of thisresearch - Indicate the form of consent obtained(written/oral)orthereasonthatconsentwasnot obtained (e.g. the data were analyzedanonymously)   **AnimalResearch(involvingvertebrateanimals,embryosortissues)**   - ProvidethenameoftheInstitutionalAnimalCare and Use Committee (IACUC) or otherrelevant ethics board that reviewed thestudy protocol, and indicate whether theyapproved this research or granted a formalwaiverofethicalapproval - Includeanapprovalnumberifonewasobtained - If the study involved *non-human primates*,add*additionaldetails*aboutanimalwelfareandstepstakentoamelioratesuffering - If anesthesia, euthanasia, or any kind ofanimalsacrificeispartofthestudy,includebriefly which substances and/or methodswereapplied   **FieldResearch**  Include the following details if this studyinvolvesthecollectionofplant,animal,orothermaterialsfromanaturalsetting:   - Fieldpermitnumber - Nameoftheinstitutionorrelevantbodythatgrantedpermission |  |
| --- | --- |
|  |  |
| **DataAvailability**  Authors are required to make all dataunderlying the findings described fullyavailable,withoutrestriction,andfromthetime of publication. PLOS allows rareexceptions to address legal and ethicalconcerns. See the [PLOS Data Policy](http://journals.plos.org/plosone/s/data-availability)and[FAQ](http://journals.plos.org/plosone/s/data-availability#loc-faqs-for-data-policy)fordetailedinformation. | Yes-alldataarefullyavailablewithoutrestriction |

| A Data Availability Statement describingwherethedatacanbefoundisrequiredatsubmission. Your answers to this questionconstitute the Data Availability Statementand **will be published in the article**, ifaccepted. |  |
| --- | --- |
| **Important:** Stating ‘data available on requestfrom the author’ is not sufficient. If your dataareonlyavailableuponrequest,select‘No’forthe first question and explain your exceptionalsituationinthetextbox. |  |
| Do the authors confirm that all dataunderlyingthefindingsdescribedintheirmanuscript are fully available withoutrestriction? |  |
| **Describe where the data may be found infull sentences. If you are copying oursampletext,replaceanyinstancesofXXXwiththeappropriatedetails.**   - If the data are **held or will be held in apublic repository**, include URLs,accession numbers or DOIs. If thisinformation will only be available afteracceptance,indicatethisbytickingthebox below. For example: *All XXX filesare available from the XXX database(accessionnumber(s)XXX,XXX.*). - Ifthedataareallcontained**withinthemanuscript and/or SupportingInformation files**, enter the following:*All relevant data are within themanuscript and its SupportingInformationfiles.* - If neither of these applies but you areable to provide **details of accesselsewhere**,withorwithoutlimitations,pleasedoso.Forexample:   *Datacannotbesharedpubliclybecauseof [XXX]. Data are available from theXXX Institutional Data Access / EthicsCommittee (contact via XXX) forresearchers who meet the criteria foraccesstoconfidentialdata.*  *The data underlying the resultspresented in the study are availablefrom(includethenameofthethirdparty* | Thedataisavailableathttps://osf.io/g6h8a/ |

| *andcontactinformationorURL).*   - This text is appropriate if the data areowned by a third party and authors donothavepermissiontosharethedata.   *typeset |  |
| --- | --- |
| Additionaldataavailabilityinformation: |  |

Manuscript	[Clickheretoaccess/download;Manuscript;PLoSOnemanuscript.docx](https://www.editorialmanager.com/pone/download.aspx?id=31394667&guid=d1230c03-ae38-4052-b563-69e859ca388a&scheme=1)

CULTURE,RUMINATION,PTSD,DEPRESSION

Does Culture Moderate the RelationshipsbetweenRuminationandSymptomsofPosttraumaticStressDisorderandDepression?

**Haoxiang Li ^1^***^¶^**, Bryan Lee ^1^***^¶^**,TamsynReyneke^1^, ShamsulHaque^2^, SitiZainab Abdullah ^2^, Britney Kerr WenTan ^2^,BelindaLiddell^3^,LauraJobson^1^**

^1^TurnerInstituteforBrainandMentalHealthandSchoolofPsychologicalSciences,MonashUniversity,Melbourne,Australia

^2^DepartmentofPsychology,JeffreyCheahSchoolofMedicineandHealthSciences,MonashUniversity,SubangJaya, Malaysia

^3^ School of Psychology, University of New South Wales, Kensington, SydneyCorrespondence:Emails:[haoxiangli@monash.edu,](mailto:haoxiangli@monash.edu)[bryanjl@student.unimelb.edu.au](mailto:bryanjl@student.unimelb.edu.au)

^¶^Theseauthorscontributedequallytothisworkandarejointfirstauthors.

Abstract

Brooding rumination is positively associated with symptoms of both depression andposttraumatic stress disorder (PTSD). However, non-clinical cross-cultural research indicatesthat culture may influence these associations. This study aimed to examine the moderatingeffect of cultural group (Australian versus Malaysian) on the associations between broodingrumination and symptoms of depression and PTSD. European Australians (*n*= 109) andMalaysians of varying Asian heritages (*n*= 144) completed an online questionnaire containingthe Hospital Anxiety and Depression Scale, PTSD checklist for DSM-5 and the RuminativeResponse Scale-Short Form. First, Malaysian participants had higher brooding ruminationthan Australian participants. Second, higher levels of brooding rumination were positivelyassociated with depression and PTSD symptom severity. Third, contrary to our expectations,culturalgroupdidnotmoderatetherelationshipsbetweenbroodingruminationandsymptoms of depression and PTSD. If replicable, these results suggest that existingassessment and treatment approaches that target brooding rumination may apply to Malaysianindividualswith depression andPTSD.

# Introduction

**Par1**Brooding rumination, defined as repetitively dwelling on the causes, consequences andmeaning of one’s negative emotions, is a transdiagnostic process associated with the etiologyand maintenance of several psychiatric disorders[1]. Posttraumatic stress disorder (PTSD)and depression are two psychiatric disorders that have received considerable attention interms of brooding rumination and symptom severity. Globally, depression and PTSD arepervasive debilitating mental health disorders, recognized in most societies and cultures, andassociatedwith numerous adverseoutcomes for affected individuals and society[2,3].

ConsideringtheprevalenceandadverseconsequencesofPTSD anddepressionworldwide,itis important to investigate factors that may contribute to these disorders and identify howculturemayinfluencesuch factors.

**Par2** Research has consistently implicated brooding rumination in the onset andmaintenance of depression. For instance, brooding rumination predicts the onset ofdepression, serves as a moderator between negative affect and depression symptoms, andmediates the relationship between several known vulnerabilities of depression and depressionsymptomatology[4-6]. The Response Styles Theory[7] posits that brooding ruminationtriggers and intensifies depression by a) prompting individuals to think negatively about theirpast, present and future, b) impeding effective problem-solving, c) inhibiting the use of goal-directedbehaviour, and d) erodingsocial support.

**Par3** Brooding rumination is also consistently associated with higher PTSDsymptomatology. Two recent meta-analyses found moderate to strong associations betweenrumination and PTSD symptoms[8, 9]. Researchers propose that that repetitively dwelling onthe negative outcomes of trauma can generate more negative trauma-related appraisals, thusincreasingarousal andanxietysymptoms[10-12].Consequently, this enhancestheassociation

between traumatic stimuli and fear and perpetuates PTSD symptoms through memorybiases[10-12].Brooding ruminationcan alsoimpedePTSDtreatment efforts[13].

**Par 4** Thus, there is considerable evidence demonstrating the relationship between broodingrumination and these two psychiatric conditions, which in turn informs current psychologicaltreatments.However,this evidencebaseis almostentirelyderivedfromWestern samples.

**Par5** Consequently, the dominant paradigm that suggests rumination is universally associated withincreasedpsychopathology,isanotionbasedlargelyonWesternresearchandculturalvalues.This is a key limitation given that (1) globally, many individuals with depression and PTSDdo not come from Western cultural backgrounds, and (2) accumulating research suggests thatthe relationship between brooding rumination and psychopathology may vary betweendifferent cultural groups.It is important to address this concerning limitation because clinicalguidelines highlight the importance of cultural tailoring in clinical practice, yet there is littleresearch to support these practices[2, 14], and treatment effects improve significantly wheninterventions are culturally-tailored[15]. Thus, it is crucial to explore how culture maymoderate the relationship between brooding rumination and psychopathology to improveculturally-tailoredtreatment approaches.

**Par 6**Accumulating research indicates that brooding rumination is more common in Asianthan Western individuals[16]. Despite this, brooding rumination has been found to be lessassociated with poor psychological outcomes, including anxiety, depression and lifedissatisfaction, in Asian individuals compared to Western individuals[17, 18]. Two keyculturaldifferences havebeenproposedtoaccountforthesefindings.First,Asiancollectivistic cultures tend to value negative emotions more favourably than Western,individualistic cultures 16, 19] and often encourage brooding rumination and self-criticism asa means of maintaining group harmony[20-22]. As such, Asian individuals may be less likelytoget ‘stuck’in theirnegativeemotions, leavingmoreroomforflexibleandadaptive

reflection[16]. Second, in Asian collectivistic cultures, when compared to Westernindividualistic cultures, there is an increased tendency to self-distance from emotionalexperiences and focus more on contextual factors[16]. Thus, thinking repetitively about thepast may be less likely to increase distress in individuals from Asian cultures. While thesestudies indicate that cultural factors may influence the psychological outcomes of broodingrumination, little research has examined cultural differences in brooding rumination inclinical populations and of the very few studies that have, all have been conducted withinWesterncountries[23].

**Paragraph 7**Additionally, cross-cultural non-clinical research in this area is limited by itssampling of mostly American and Chinese populations. Australia and Malaysia are twoheavily underrepresented populations. While Australia and America are both portrayed asindividualistic societies[24], Australians have lower autonomy needs than Americans andmay not be as individualistic as America[25]. Similarly, Malaysia is a multi-cultural societyprimarily populated by individuals of Malay, Chinese and Indian heritage[26]. Like China,Malaysia is considered to be collectivistic[26]. However, the presence of other culturalfactors within Malaysian societies may influence their social emphasis on collectivism[27].For instance, unlike Chinese cultures which base their collectivisms on Confucianism,Malaysians also obtain their emphasis on collectivism from the traditional cultures of Islamand the indigenous Malay[27]. Hence, it is crucial to explore the relationships betweenbrooding rumination and psychological adjustment in varying Western individualistic andAsiancollectivistic cultures.

**Paragraph 8**This study aimed to investigate the moderating effect of cultural group on theassociation between brooding rumination and symptoms of PTSD and depression within anAsian collectivistic (Malaysia) and Western individualistic (Australia) cultural context. Wehypothesizedthat therewouldbegreaterbroodingrumination reported bythe Malaysian

group than the Australian group (Hypothesis 1). Second, brooding rumination would bepositively associated with both depression and PTSD symptom severity (Hypothesis 2).Third, cultural group would moderate the associations between brooding rumination and bothdepression and PTSD symptoms, such that brooding rumination would be more stronglyassociated with symptom severity in the Australian group than Malaysian group (Hypothesis3).

# Method

## Participants

**Paragraph 1:**Given the prevalence of depression and trauma-exposure in the general community, a general community sample provided a wide range of symptomatology allowing for thehypothesized relationships to be examined. Utilising G*Power[28], with a small-moderateeffect size (*f*^2^= .14)[29], an alpha of .05 and power of .80, 101 participants (50 per culturalgroup)werefoundtobe sufficienttopower allhypotheses.Usingconveniencesampling,253participants(109Australian,144Malaysian) were recruitedfromthe generalcommunity.

**Paragraph 2:**Eligibility criteria included being a) between 18 and 65 years of age, b) able to read andcomplete the questionnaire in English or Malay, and c) either residing in Australia and identifying as having European heritage or residing in Malaysia and identifying as havingeitherMalay,IndianorChineseheritage.Additionally,tobeincludedinthePTSDanalyses,participants had to have been exposed to a Criterion A trauma experience as indexed on theLifeEvents Checklist-5[30](Australian *n*=107,Malaysian*n* =121).

## Measures

**Hospital Anxiety and Depression Scale (HADS).** The depression subscale of theHADS (HADS-D)[31] was used to measure depression symptoms. The subscale containsseven items scored on 4-point Likert-type scales (0 = *not at all* to 3 = *definitely*). Individualitemscoresweresummed (fiveitemsarereversedscored)togive atotaldepressionscore

rangingfrom0to21,withhigherscoresindicatinggreaterdepressionsymptomseverity.Thismeasure has demonstrated good validity and reliability, including in cross-culturalresearch[32]. In this study the HADS-D demonstrated acceptable internal consistency(Australiaα=.78, Malaysiaα =.80).

**PTSDChecklistforDSM-5(PCL-5)withLifeEventsChecklistforDSM-5(LEC-5).**The PCL-5 with LEC-5[30] assessed trauma exposure and PTSD symptom severity. TheLEC-5screensforexposuretotraumaticeventsinarespondent'slifetime.Participantsidentifytheir worst single traumatic event; the *index trauma*. The LEC-5 is not scored, but used toensure trauma exposure (i.e., eligibility criteria) and categorise participants’ index traumas.The PCL-5 checklist is a 20-item self-report questionnaire that assesses PTSD symptomseverity in relation to the respondent’s index trauma. Items are scored using 5-point Likertscales (0 = *not at all* to 4 = *extremely*). Scores are summed to yield a total score ranging from0-80,withhigherscoresindicatinggreaterPTSDsymptomseverity.ThePCL-5hasdemonstrated good psychometric properties, including in cross-cultural research[33]. In thepresentstudy, internalconsistencywas excellent(Australian α=.95; Malaysian α =.96).

**Ruminative Response Scale-Short Form (RRS-SF).**Brooding rumination wasmeasured using the brooding subscale of the RRS-SF (RRS-B)[34]. The five items werescored on 4-point Likert-type scales (1 = *almost never* to 4 = *almost always*). Item scores aresummed and range from 5 to 20, with higher scores indicating greater levels of broodingrumination. The RRS-B has been shown to have good reliability and validity, including incross-cultural research[35]. The RRS-B demonstrated good internal consistency in this study(Australianα=.79, Malaysian α=.84).

**Conscientious Responders Scale (CRS).** The CRS was utilised to identifyparticipants who answered questions hastily and inaccurately[36]. The five items wereinterspersedthroughoutthequestionnaire.Eachitem directlyinstructsparticipants onhowto

answer and is scored as either correct (1) or incorrect (0). Scores ranged from 0-5, andparticipantswhoscoredless than3on theCRSwereexcludedfromdataanalyses,basedonless than 3% of random responders achieving a score of 3 or above by chance alone[36,37].**Procedure**

Ethical approval was obtained from BLINDED. To ensure cultural appropriateness,the study was co-designed by Australian and Malaysian researchers. Following the goldstandard approach[38], English measures were translated into Malay and then back-translatedinto English. The study was advertised on social media (Facebook and Gumtree). Interestedrespondentscontacted theresearchers via email and received alinktotheonlinequestionnaire (Qualtrics). Participants completed the PCL-5 with LEC-5, HADS-D, RRS-Banddemographics. Participants werereimbursedwith gift vouchers.

## StatisticalAnalyses

All statistical analyses were conducted using SPSS (version 27.0). As some variablesdid not meet assumptions for normality, bootstrapping was utilised for all analyses. Givensignificant group differences in age, education and religion, these variables were included ascovariates. A subset of the sample (*n* = 228) was included in the PTSD analyses; to beincluded in the PTSD analyses participants had to have been exposed to a Criterion A traumaexperience.Totest Hypothesis1,a one-wayanalysis ofcovariancewasconductedcomparingthe Malaysian and Australian groups with brooding rumination as the dependent variable. Toassess Hypothesis 2, Spearman’s correlation analysis examining the associations betweenbrooding rumination, depression and PTSD symptoms were conducted in the overall sample,and for each separate group. To test Hypothesis 3, two moderation analyses (model 1) usingPROCESS v3.5[39] were conducted to determine whether cultural group moderated theassociationsbetween1)broodingruminationanddepressionsymptoms,and 2) brooding

rumination and PTSD symptoms. Significance of results was indicated by 95% confidenceintervalsnot includingzero.

# Results

Table 1 presents participant characteristics and shows significant group differencesforage,education, religion andtime sincetrauma.

## Hypothesis 1

As shown in Table 1, Malaysian participants had significantly greater broodingrumination thanAustralianparticipants,*F*(1, 228)= 5.77,*p*=.02,𝜂^2^= .03.

𝑝

## Hypothesis 2

There was a statistically significant, moderate positive correlation between broodingrumination and depression, *rs*(251) =.42, 95%CI [.29, .52], *p* < .001, and between PTSD andbroodingrumination, *rs*(226)=.51, 95%CI[.40, .60],*p* <.001. Whenexaminingeachculturalgroup separately, there was a statistically significant positive associations between broodingrumination and depression in the Malaysian, *rs*(142) =.50, 95%CI [.36, .62], *p* < .001, andAustraliangroups,*rs*(107)=.31,95%CI[.10,.50],*p*=.001,andbetweenbroodingrumination and PTSD in the Australian, *rs*(105) =.43, 95%CI [.26, .57], *p* < .001 andMalaysian groups, *rs*(119) =.59, 95%CI [.45, .70], *p* < .001. The PTSD findings were similarwhentrauma typeandtime sincetraumawereincluded as covariates.

## Hypothesis 3

Table 2 presents the results of the moderation analyses. There was no evidence thatcultural group moderated the associations between brooding rumination and depression, *F*(1,249) = 3.03, *p* = .08, R^2^Δ = .01, or brooding rumination and PTSD symptoms, *F*(1, 220) =2.06, *p* = .15, R^2^Δ < .01. The PTSD findings were similar when trauma type and time sincetraumawereincludedascovariates

# Discussion

Thisstudyinvestigatedthemoderating effectofculturalgroup(AustraliavsMalaysia) on the relationships between brooding rumination and symptoms of depression andPTSD. In support of Hypothesis 1, we found that Malaysian participants had higher levels ofbrooding rumination compared to Australian participants. In support of Hypothesis 2,brooding rumination was significantly associated with both greater depression and PTSDsymptom severity. Inconsistent with Hypothesis 3, we did not find a significant moderatingeffect of culturalgroup on therelationship betweenbroodingrumination andeitherdepressionorPTSD symptom severity.

Our finding that Malaysians reported higher levels of brooding rumination comparedto Australians aligns with previous cross-cultural non-clinical research; members ofcollectivistic cultures (e.g., Malaysia) tend to brood more than members of individualisticcultures (e.g., Australia)[18,29,40]. In relation to Hypothesis 2, we found that broodingrumination was positively associated with both depression and PTSD symptom severity inboth Australian and Malaysian groups, which also aligns with previous literature indicatingthat broodingruminationis associated with increased psychopathology[9,41].

However, there was no evidence of a moderating effect of cultural group on therelationship between brooding rumination and either symptoms of PTSD or depression. Thisobservation does not align with the expected cross-cultural differences theorized andobserved in non-clinical research which indicates brooding rumination is less associated withpsychopathology for Asian individuals than Western individuals[16-18]. Our results suggestthat these cultural differences observed in the non-clinical literature may not extend toclinical populations. It is possible that at higher levels of psychopathology, the strongnegative effect of brooding rumination may override the cultural differences found in healthypopulations.Such anotion isconsistent withexistingliteraturewhichimplicates highlevels

of brooding rumination as a transdiagnostic maintenance factor across many disorders[41],which may play a role regardless of cultural background[23]. Thus, when consideringpsychopathology, broodingruminationmaybesimilarlymaladaptive.

If such findings are robust and replicable across other cultures, existing clinicalmodels, which implicate brooding rumination in the maintenance of depression and PTSD(e.g., [7,11]), may be applicable cross-culturally. Additionally, as existing evidence-basedtreatments for depression and PTSD recommend brooding rumination as a keytarget[8,42,43],suchatarget maybesuitablefor Malaysianclients.However,futureresearchis needed to further understand the content and form of brooding rumination in Malaysianclinical populations and the influence of culture on the role of brooding rumination inpsychopathology.

The study had several limitations. First, we did not measure participants’ culturalviews and beliefs, and instead utilised nationality (Australian or Malaysian) and culturalheritage as an indicator of cultural orientation. While this approach was consistent withprevious cross-cultural research[29,44], future research could also examine cultural values atthe individual level[45]. Second, we did not utilise a clinical sample. Third, the cross-sectional study design does not allow for causal inferences to be made. Finally, there weresignificantly more females than males in our study. Females tend to ruminate more on theirdepressive symptoms[46,47]. Consequently, the mean levels of brooding rumination anddepressionsymptomscouldhave beeninflatedbytheimbalancedgenderratiosofparticipants in the study. Despite these limitations, we found Malaysians had higher levels ofbroodingrumination than Australians, and thatbroodingruminationwaspositivelyassociatedwithPTSD and depression symptom severityin both cultural groups.

# Acknowledgments

Aspecialthanks toall theparticipants thatentered thestudy.

# DataAvailability Statement

Dataisavailableathttps://osf.io/g6h8a/

# References

1. Johnson DP, Rhee SH, Friedman NP, Corley RP, Munn-Chernoff MA, Hewitt JK,et al. A twin study examining rumination as a transdiagnostic correlate ofpsychopathology. Clinical Psychological Science [Internet]. 2016 Nov 1 [cited2022 Jun 22];4(6):971–87. Available from:https://discovery.ebsco.com/linkprocessor/plink?id=2ed20b76-ae50-3699-9a63-c4e6c9e7b96d
2. *American Psychiatric Association. Diagnostic and statistical manual of mentaldisorders.*5^th^ed.American PsychiatricAssociation Publishing; 2022.
3. World Health Organization. Depression fact sheet [Internet]. World HealthOrganization; 2020 [updated 13 Sep 21; cited 2022 Jun 22]. Available from:https:/[/www.who.int/news](http://www.who.int/news-room/fact-sheets/detail/depression)-[room/fact-sheets/detail/depression](http://www.who.int/news-room/fact-sheets/detail/depression)
4. KrauseED,VélezCE,WooR,HoffmannB,FreresDR,AbenavoliRM,etal.

Rumination,depression,and genderinearlyadolescence: Alongitudinal studyof abidirectional model. J Early Adolesc [Internet]. 2018 [cited 2022 Jun22];38(7):923–46.Availablefrom:<http://dx.doi.org/10.1177/0272431617704956>

5. Verstraeten K, Bijttebier P, Vasey MW, Raes F. Specificity of worry andrumination in the development of anxiety and depressive symptoms in children:Specificity of worry and rumination. Br J ClinPsychol [Internet]. 2011 [cited 2022Jun 22];50(4):364–78. Available from:<http://dx.doi.org/10.1348/014466510X532715>
6. Yoon KL, Maltby J, Joormann J. A pathway from neuroticism to depression:examiningtheroleofemotionregulation.AnxietyStressCoping[Internet].2013[cited 2022 Jun 22];26(5):558–72. Available from:<http://dx.doi.org/10.1080/10615806.2012.734810>
7. Nolen-Hoeksema S. Responses to depression and their effects on the duration ofdepressiveepisodes. JAbnormPsychol [Internet]. 1991[cited 2022 Jun22];100(4):569–82.Availablefrom:<http://dx.doi.org/10.1037/0021-843x.100.4.569>
8. Moulds ML, Bisby MA, Wild J, Bryant RA. Rumination in posttraumatic stressdisorder: A systematic review. ClinPsychol Rev [Internet]. 2020 [cited 2022 Jun22];82(101910):101910. Available from:<http://dx.doi.org/10.1016/j.cpr.2020.101910>
9. Seligowski AV, Lee DJ, Bardeen JR, Orcutt HK. Emotion regulation andposttraumatic stress symptoms: a meta-analysis. CognBehavTher [Internet]. 2015[cited 2022 Jun 22];44(2):87–102. Available from:<http://dx.doi.org/10.1080/16506073.2014.980753>
10. Dalgleish T. Cognitive approaches to posttraumatic stress disorder: the evolution ofmultirepresentational theorizing. Psychological Bulletin [Internet]. 2004 Mar 1[cited 2022 Jun 22];130(2):228–60. Available from:https://discovery.ebsco.com/linkprocessor/plink?id=0313daff-2821-31f4-9559-03cbefcf392a
11. EhlersA,ClarkDM.A cognitivemodelofposttraumaticstressdisorder.BehaviourResearch and Therapy [Internet]. 2000 Jan 1 [cited 2022 Jun 22];38(4):319–45.Available from: https://discovery.ebsco.com/linkprocessor/plink?id=ec694699-ded7-3f36-8cc2-dc5f559a98eb
12. WilliamsJMG,BarnhoferT,CraneC,Herman D, RaesF,WatkinsE,etal.

Autobiographical memory specificity and emotional disorder.Psychol Bull[Internet]. 2007 [cited 2022 Jun 22];133(1):122–48. Available from:<http://dx.doi.org/10.1037/0033-2909.133.1.122>

13. Effective treatments for PTSD: practice guidelines from the International Societyfor Traumatic Stress Studies / edited by Edna B. Foa ... [and others] [Internet].Guilford Press; 2009 [cited 2022 Jun 22]. Available from:https://discovery.ebsco.com/linkprocessor/plink?id=d01f4aab-768b-3ba1-aebc-0706acd571ba
14. Bernal G, Saez-Santiago E. Culturally centered psychosocial interventions. Journalof CommunityPsychology[Internet]. 2006 Mar 1[cited 2022 Jun 22];34(2):121–

32. Available from: https://discovery.ebsco.com/linkprocessor/plink?id=7f38362f-23c1-31db-9729-726d81beefb9

15. Huey Jr. SJ, Tilley JL. Effects of mental health interventions with AsianAmericans: a review and meta-analysis. Journal of Consulting & ClinicalPsychology [Internet]. 2018 Nov 1 [cited 2022 Jun 22];86(11):915–30. Availablefrom: https://discovery.ebsco.com/linkprocessor/plink?id=3a648e28-ee28-3053-b35b-d1bb95d155cf
16. De Vaus J, Hornsey MJ, Kuppens P, Bastian B. Exploring the East-West divide inprevalence of affective disorder: a case for cultural differences in coping withnegative emotion. Personality and social psychology review [Internet]. 2018 Jan 1[cited 2022 Jun 22];22(3):285–304. Available from:https://discovery.ebsco.com/linkprocessor/plink?id=97416cea-df74-3bd0-a7dc-36f843e537f0
17. Bonanno GA, Papa A, Lalande K, Zhang N, Noll JG. Grief processing anddeliberate grief avoidance: a prospective comparison of bereaved spouses andparents in the United States and the People’s Republic of China. Journal ofConsulting & Clinical Psychology [Internet]. 2005 Feb 1 [cited 2022 Jun22];73(1):86–98.Available from:

https://discovery.ebsco.com/linkprocessor/plink?id=9f3d6d2c-dfbc-3401-88bb-441818c9ff80

18. Chang EC, Tsai W, Sanna LJ. Examining the relations between rumination andadjustment:doethnicdifferencesexistbetweenAsianandEuropeanAmericans.Asian American Journal of Psychology [Internet]. 2010 Mar 1 [cited 2022 Jun22];1(1):46–56. Available from:https://discovery.ebsco.com/linkprocessor/plink?id=c2d5b2e7-3afb-3fdd-b9b4-1d89c62ffd08
19. Zhang M, Cross SE. Emotions in memories of success and failure: a culturalperspective. Emotion [Internet]. 2011 [cited 2022 Jun 22];11(4):866–80. Availablefrom:<http://dx.doi.org/10.1037/a0024025>
20. FryPS,GroverSC.Cognitiveappraisalsoflifestressanddepressionintheelderly:a cross-cultural comparison of Asians and Caucasians. International Journal ofPsychology [Internet]. 1982 Dec 1 [cited 2022 Jun 22];17(4):437. Available from:https://discovery.ebsco.com/linkprocessor/plink?id=b9934d22-055d-3c2c-b8e8-389544829b14
21. Kitayama S, Markus HR, Matsumoto H, Norasakkunkit V. Individual and collectiveprocesses in the construction of the self: self-enhancement in the United States andself-criticism inJapan.Journalof PersonalityandSocialPsychology[Internet].1997 Jan 1 [cited 2022 Jun 22];72(6):1245–67. Available from:https://discovery.ebsco.com/linkprocessor/plink?id=55a084b1-cc3c-3b02-b10c-9716729822fb
22. Nisbett RE, Peng K, Choi I, Norenzayan A. Culture and systems of thought: holisticversusanalyticcognition.Psychol Rev [Internet].2001[cited 2022Jun

22];108(2):291–310. Available from: <http://dx.doi.org/10.1037//0033->295x.108.2.291

23. Nagulendran A, Jobson L. Exploring cultural differences in the use of emotionregulation strategies in posttraumatic stress disorder. Eur J Psychotraumatol[Internet]. 2020 [cited 2022 Jun 22];11(1):1729033. Available from:<http://dx.doi.org/10.1080/20008198.2020.1729033>
24. Church A, Alvarez JM, Katigbak MS, Mastor KA, Cabrera HF, Tanaka-Matsumi J,et al. Self-concept consistency and short-term stability in eight cultures. Journal ofResearch in Personality [Internet]. 2012 Oct 1 [cited 2022 Jun 22];46(5):556–70.Available from: https://discovery.ebsco.com/linkprocessor/plink?id=a352b519-abd6-39e4-b81a-7ada5637b058
25. Church T, Katigbak MS, Locke KD, Zhang H, Shen J, de Jesus Vargas-Flores J, etal. Need satisfaction and well-being: testing self-determination theory in eightcultures. Journal of Cross Cultural Psychology [Internet]. 2013 Jan 1 [cited 2022Jun 22];44(4):507–34. Available from:https://discovery.ebsco.com/linkprocessor/plink?id=aca452c1-f5a1-3091-9346-70c5dcb19081
26. TanCB.Ethnicidentitiesandnationalidentities:someexamplesfromMalaysia.

Identities [Internet]. 2000 Jan 1 [cited 2022 Jun 22];6(4):441–80. Available from:https://discovery.ebsco.com/linkprocessor/plink?id=e87f46ec-f61e-3bdd-97b8-ebad9ab73604

27. Chang WC, Osman MMB, Tong EMW, Tan D. Self-construal and subjective well-being in two ethnic communities in Singapore. Psychology [Internet]. 2011 April 2[cited 2022 Jun 22]; 2*(*2):63–70. Available from:https:/[/www.scirp.or](http://www.scirp.org/journal/paperinformation.aspx?paperid=4426)g[/journal/paperinformation.aspx?paperid=4426](http://www.scirp.org/journal/paperinformation.aspx?paperid=4426)
28. Faul F, Erdfelder E, Lang A-G, Buchner A. G*Power 3: a flexible statistical poweranalysis program for the social, behavioral, and biomedical sciences. Behav ResMethods [Internet]. 2007 [cited 2022 Jun 22];39(2):175–91. Available from:<http://dx.doi.org/10.3758/bf03193146>
29. Grossmann I, Kross E. The impact of culture on adaptive versus maladaptive self-reflection. Psychological Science [Internet]. 2010 Aug 1 [cited 2022 Jun22];21(8):1150–7. Available from:https://discovery.ebsco.com/linkprocessor/plink?id=7cf79047-9384-3930-ad81-e32aad7d6af8
30. Blevins CA, Weathers FW, Davis MT, Witte TK, Domino JL. The PosttraumaticStress Disorder Checklist for DSM-5 (PCL-5): development and initialpsychometric evaluation. J Trauma Stress [Internet]. 2015 [cited 2022 Jun22];28(6):489–98.Availablefrom:https://pubmed.ncbi.nlm.nih.gov/26606250/
31. ZigmondAS,SnaithRP.Thehospital anxietyanddepressionscale.ActaPsychiatrScand [Internet]. 1983 [cited 2022 Jun 22];67(6):361–70. Available from:<http://dx.doi.org/10.1111/j.1600-0447.1983.tb09716.x>
32. Li Q, Lin Y, Hu C, Xu Y, Zhou H, Yang L, et al. The Chinese version of hospitalanxiety and depression scale: psychometric properties in Chinese cancer patientsandtheirfamilycaregivers. EuropeanJournalofOncologyNursing[Internet].2016Dec 1 [cited 2022 Jun 22];25:16–23. Available from:https://discovery.ebsco.com/linkprocessor/plink?id=fc3575aa-782e-32a2-9289-6809972b3e0e
33. Sveen J, Bondjers K, Willebrand M. Psychometric properties of the PTSDChecklist for DSM-5: a pilot study. Eur J Psychotraumatol [Internet]. 2016 [cited2022Jun22];7(1):30165.Availablefrom:<http://dx.doi.org/10.3402/ejpt.v7.30165>
34. Treynor W, Gonzalez R, Nolen-Hoeksema S. Rumination reconsidered: apsychometric analysis. Cognitive Therapy and Research [Internet]. 2003 Jun 1[cited 2022 Jun 22];27(3):247–59. Available from:https://discovery.ebsco.com/linkprocessor/plink?id=97bcef6c-e224-35ee-b194-34d1898b1fa2
35. Lee S, Kim W. Cross-cultural adaptation, reliability, and validity of the revisedKorean version of Ruminative Response Scale. Psychiatry Investigation [Internet].2014 Jan 1 [cited 2022 Jun 22];11(1):59–64. Available from:https://discovery.ebsco.com/linkprocessor/plink?id=c62f6c2e-3850-31f2-8e02-8ba03b29e07d
36. Marjanovic Z, Struthers CW, Cribbie R, Greenglass ER. The ConscientiousRespondersScale: anew toolfordiscriminatingbetweenconscientiousandrandomresponders.SAGEOpen[Internet]. 2014[cited2022 Jun22];4(3):215824401454596. Available from:<http://dx.doi.org/10.1177/2158244014545964>
37. MarjanovicZ, Bajkov L, MacDonaldJ.TheConscientiousRespondersScalehelpsresearchers verify the integrity of personality questionnaire data. Psychol Rep[Internet]. 2019 [cited 2022 Jun 22];122(4):1529–49. Available from:<http://dx.doi.org/10.1177/0033294118783917>
38. Epstein J, Santo RM, Guillemin F. A review of guidelines for cross-culturaladaptation of questionnaires could not bring out a consensus. Journal of ClinicalEpidemiology [Internet]. 2015 Apr 1 [cited 2022 Jun 22];68(4):435–41. Availablefrom: https://discovery.ebsco.com/linkprocessor/plink?id=8c2afddc-feb0-3b33-abd9-a568ed16508a
39. HayesAF. Introductionto mediation,moderation,andconditionalprocess analysis:a regression-based approach / Andrew F. Hayes [Internet]. The Guilford Press;2013 [cited 2022 Jun 22]. Available from:https://discovery.ebsco.com/linkprocessor/plink?id=553e185b-1b86-3781-a932-e5ae8f393391
40. Kwon H, Yoon KL, Joormann J, Kwon J-H. Cultural and gender differences inemotion regulation: relation to depression. Cognition & Emotion [Internet]. 2013Aug 1 [cited 2022 Jun 22];27(5):769–82. Available from:https://discovery.ebsco.com/linkprocessor/plink?id=4ff92515-9df7-3d5d-80ac-900903270aaa
41. McLaughlin KA, Nolen-Hoeksema S. Rumination as a transdiagnostic factor indepression and anxiety. Behav Res Ther [Internet]. 2011 [cited 2022 Jun22];49(3):186–93.Availablefrom:<http://dx.doi.org/10.1016/j.brat.2010.12.006>
42. AhernE,KinsellaS,SemkovskaM.Clinicalefficacyandeconomic evaluationofonline cognitive behavioral therapy for major depressive disorder: a systematicreview and meta-analysis. Expert review of pharmacoeconomics& outcomesresearch [Internet]. 2018 Jan 1 [cited 2022 Jun 22];18(1):25–41. Available from:https://discovery.ebsco.com/linkprocessor/plink?id=6cc03ebe-9add-35a7-b882-4b0b57195b0c
43. Querstret D, Cropley M. Assessing treatments used to reduce rumination and/orworry: a systematic review. ClinPsychol Rev [Internet]. 2013 [cited 2022 Jun22];33(8):996–1009.Availablefrom:<http://dx.doi.org/10.1016/j.cpr.2013.08.004>
44. Mihailova S, Jobson L. The impact of depression and culture on responses tointrusiveautobiographicalmemories:cognitiveappraisals,cognitiveavoidance,and

brooding rumination. Br J ClinPsychol [Internet]. 2020 [cited 2022 Jun22];59(1):66–79.Availablefrom:<http://dx.doi.org/10.1111/bjc.12232>

45. Oyserman D, Coon HM, Kemmelmeier M. Rethinking individualism andcollectivism:evaluationoftheoreticalassumptionsandmeta-analyses.PsycholBull[Internet]. 2002 [cited 2022 Jun 22];128(1):3–72. Available from:<http://dx.doi.org/10.1037/0033-2909.128.1.3>
46. Nolen-Hoeksema S, Aldao A. Gender and age differences in emotion regulationstrategiesandtheirrelationshiptodepressivesymptoms.PersIndividDif[Internet].2011 [cited 2022 Jun 22];51(6):704–8. Available from:<http://dx.doi.org/10.1016/j.paid.2011.06.012>
47. Salk RH, Hyde JS, Abramson LY. Gender differences in depression inrepresentative national samples: meta-analyses of diagnoses and symptoms.Psychol Bull [Internet]. 2017 [cited 2022 Jun 22];143(8):783–822. Available from:<http://dx.doi.org/10.1037/bul0000102>

## Table1.ParticipantCharacteristics

AustralianGroup	MalaysianGroup	Statistic

| Age(years) | 31.53(12.68) | 25.88(7.64) | *t*(165.2)=4.06^**^ |
| --- | --- | --- | --- |
| Gender^a^ | 21:87:1 | 32:108:4 | χ^2^(2,*N*=253)=1.53 |
| Education^b^ | 27:21:39:20:2 | 12:10:94:20:8 | χ^2^(4,*N*=253)=31.78^**^ |
| Religion^c^ | 36:57:7:0:0:7:2 | 3:22:48:37:18:7:9 | χ^2^(5,*N*=253)=127.46^**^ |
| HADS-D | 5.8(3.76) | 5.65(3.83) | *t*(251)=.30 |
| Index trauma^d,e^ | 35:15:15:28:8:6 | 49:17:12:21:14:8 | χ^2^ (6,228)=6.26 |
| Timesincetrauma^d^ | 10.18(12.38) | 5.87(5.75) | *t*(146)=3.30^**^ |
| PCL-5^d^ | 22.96(17.68) | 22.69(18.45) | *t*(226)=.12 |
| RRS-B | 11.32(3.69) | 11.54(3.7) | *F*(1,228)=5.77* |

*Note*.Datashownisthe mean(SD)forallcharacteristicsexceptgender, religionand

education* *p* <.05 ** *p*<.01

HADS-D = Hospital Anxiety and Depression Scale (Depression subscale); PCL-5 = PTSDChecklistforDSM-5; RRS-B=RuminativeResponse Scale(Broodingsubscale)

^a^Male: female: prefer notto say

^b^ Self-reported highest education level attained: secondary education: post-secondaryeducation:undergraduatedegree: postgraduate degree:other

^c^Self-reportedreligiousbackground:noreligion: Christianity:IslamorMuslim:BuddhismorTaoism:Hinduism:Other(includingSikhismandAgnosticism):didnotrevealreligion^d^ForthePTSD data onlydata from asubset of theoverallsamplewas used.

^e^Indextraumacategory,displayedasaccident,seriousinjuryorillness:non-sexualassaultorabuse:sexual assault: witnessingdeath:war or natural disaster: other.

## Table2.SummaryofModerationAnalyses

Model	Coefficient	*t	p*

|  | | *B* | SE | [95%CI] |  | |
| --- | --- | --- | --- | --- | --- | --- |
| Depression | Constant | 5.60 | 0.29 | [5.04-6.16] | 19.63 | < .001 |
|  | RRS-Bscore | 0.53 | 0.08 | [.38-.68] | 6.83 | < .001 |
|  | Culturalgroup | 0.24 | 0.43 | [-.62-1.09] | 0.54 | .59 |
|  | Interaction | -0.21 | 0.12 | [-.44-.03] | -1.74 | .08 |
| PTSD | Constant | 23.20 | 5.68 | [11.82,34.49] | 4.17 | < .001 |
|  | RRS-Bscore | 3.77 | 0.97 | [1.84, 5.64] | 4.41 | < .001 |
|  | Culturalgroup | 0.63 | 2.39 | [-3.89,5.38] | 0.25 | .80 |
|  | Interaction | -0.81 | 0.64 | [-2.07,0.40] | -1.43 | .15 |

*Note*:PTSD=PosttraumaticStressDisorder. RRS-BRuminativeResponse Scale(Broodingsubscale)
