# Supplementary material for: Does culture moderate the relationships between rumination and symptoms of posttraumatic stress disorder and depression?
Source: PLoS One. 2022 Nov 29;17(11):e0278328. doi: 10.1371/journal.pone.0278328 (PMC9707787; doi:10.1371/journal.pone.0278328)
Supplement: Attachment — Submitted filename: Review report (1).docx [file pone.0278328.s003.docx]

**Review Report**

**PLOSONE**

**DoesCultureModeratetheRelationshipsbetweenRuminationandSymptomsofPosttraumaticStressDisorderandDepression?**

**ManuscriptNumber:** PONE-D-22-19386

**ArticleType:** ResearchArticle

Reviewer’sname:

Institution:

Date received: 16/05/2022

# Date returned: 08/06/2022

**General Comment**

Generally, the paper is original and can be published given strong comments editors. However, the paper needs a lot of revisions in terms of flow of ideas, contents, etc. I tried to highlight the issues that should be addressed in-text of the paper. In addition, I highlighted the main issues section by section to be addressed by the authors as follows.

**Title:**

**“*DoesCultureModeratetheRelationshipsbetweenRuminationandSymptomsofPosttraumaticStressDisorderandDepression?*”**

- This title likely deservesa binary response “Yes/No”. Then, what will happen after “Yes/No” response? It seems the last answer to the research question. I strongly recommend that the authors should avoid interrogation in writing the title of the paper. Instead, they can rewrite it affirmative sentence as follows:

***“The Role of Culturein ModeratingtheRelationshipsbetweenRuminationandSymptomsofPosttraumaticStressDisorderandDepression*”**

Or,

Using what is provided as a short title would be sufficient: ***culturaldifferencesinruminationindepressionandPTSD***(in expanded form).

**Abstract:**

- The first sentence is like your research finding. In practice however, you should start with brief introduction to the problem (may be in a sentence).
- As long as the contents of “**Abstract**” contains the following:
- Start with brief introduction to the problem/title
- State the objective in brief,
- State the methods and materials used in brief,
- Provide the main findings in brief,
- Give conclusion in brief and recommendation (if necessary)

This is sufficient for addressing Abstract section.

- In the Keywords, avoid one of the words (PTDS or post-traumaticstressdisorder) and use one of them.

**Introduction**

**Paragraph 1:**attempts to elaborate that the authors will focus on culture as only factor that causes thedifferences in the RelationshipsbetweenRuminationandSymptomsofPosttraumaticStressDisorderandDepression. This leads the reader to understand that the only factor causing differences in the RelationshipsbetweenRuminationandSymptomsofPosttraumaticStressDisorderandDepression. However, many more factors are responsible for such differences. So, the finding you arrived at and the conclusion drawn may not be reliable unless you could statistically capture the extraneous variables/factor that may also be responsible for such differences. So, How can you harmonize this gap between your ‘culture only variable” and “the impacts of other non-cultural variables”? To conclude whatever it is, you should be able to tell reader how you managed the influence of the other factor than culture. Otherwise, your conclusion may not be correct.

**Paragraph6:**The overall idea of this paragraph is that cultural differences in brooding rumination has been conducted but not in the study area because such studies are concentrated in western countries but not in Australia. Well, for one thing the authors must exhaustively reviewed literature to be sure of this. Second, the idea that previous literature established the theory that there is such cultural difference in brooding rumination can also apply to many other cultures. As a result, the authors may be asked what the need to conduct the research in Australia is. They need to justify this very clearly.

**Paragraph 8:**This paragraph states different hypotheses. This is good but your topic is only on Yes/No type. However, here your hypotheses themselves talk much more than the binary responses that your topic informs the reader. So, rewrite your topic in line with these hypotheses.

# Method

Generally, this section is addressed well. However, some seemingly minor but can introduce significant problem to the paper are observed. For example, The authors mentioned no research design employed. Instead, they began with presenting the participants. What is the specific research design for this study? The authors should elaborate a bit about the basic research design immediately after this section. In my opinion, your research design is ‘cross-sectional survey”. Therefore, you would have explained how and why you employed the design before providing your sampling method.

**Paragraph 1:**Was Convenience sampling or snowball sampling appropriate? This because the method by which the researchers accessed the respondents was via internet system (facebook&Gumtree). Even, the method you used seems different from snowballing and convenience sampling. I suggest strongly to re-describe this method appropriately.

Moreover, *General community sample* in the same paragraph is not clear as you cannot find it in a scientific circle. It seems your creation, otherwise try to mention some literatures that use similar phrase. This type of sampling is not known by the scientific community. Instead, elaborate the sampling technique you employed for the study…. In case how you employed a convenience sampling procedure although whether you employed convenience sampling method or not is still unclear.

**Paragraph 2:** I think the phrase ‘eligibility criteria’ is to mean inclusion and exclusion criteria. It is okay; but you have no reason to limit the age range of the respondents. Incase you thought that you selected those who can read and fill out the questionnaire, at present children who completed their primary education can do that. Therefore, you should justify for limiting the age range you mentioned here.

**Results**

Giving the result precisely is not bad since all hypotheses are covered.

**Discussion**

The serious problem lies here. This section is too shallow to draw the conclusion. The authors did not discussed their findings with sufficient number of related previous literature. They mainly focused on how the findings against each hypothesis agrees or disagrees each other. I recommend the authors should discuss further their findings in relation to previous literature in depth.
